# Immunological Effects of Environmental Factors: Focus on the Fibrous and Particulated Materials

**DOI:** 10.1155/2014/697438

**Published:** 2014-12-21

**Authors:** Takemi Otsuki, Andrij Holian, Mario Di Gioacchino

**Affiliations:** ^1^Department of Hygiene, Kawasaki Medical School, Kurashiki, Okayama 7010192, Japan; ^2^Center for Environmental Health Sciences, University of Montana, Missoula, MT 59812, USA; ^3^Immunotoxicology and Allergy Unit, Ce.S.I., “G. d'Annunzio” University Foundation, Department of Medicine and Ageing Science, “G. d'Annunzio” University, 66100 Chieti, Italy

On behalf of guest editors for this special issue, we are very pleased to publish this special interest issue. The immunological effects of various fibrous and particulate materials such as asbestos and silica as well as nanoparticles and nanotubes have received increased attention in recent years. These materials are now well known for their biological effects that include lung fibrosis and carcinogenic potential, which is the basis for the scientific interest for environmental health. Furthermore, the immunological effects of these substances are also very important issues since, from the point of initial administration of these materials, the innate immune system will recognize these materials as foreign danger signals and they may affect the systemic immune system. In this special issue, various recent investigations regarding the above-mentioned viewpoints will provide readers with recent advances in the area of environmental immunology. We hope that this special issue will contribute to the better understanding and considerations for biological effects of fibrous and particulate materials, particularly on the human immune systems.

*Takemi Otsuki* *Takemi Otsuki* *Andrij Holian* *Andrij Holian* *Mario Di Gioacchino* *Mario Di Gioacchino*

